# Cyclophilin A: promising target in cancer therapy

**DOI:** 10.1080/15384047.2024.2425127

**Published:** 2024-11-08

**Authors:** Shujuan Jin, Mengjiao Zhang, Xiaoting Qiao

**Affiliations:** a Shenzhen Institute for Technology Innovation, National Institute of Metrology, Shenzhen, Guangdong, China; b Chenxi Women’s and Children’s Hospital, Huaihua, Hunan, China

**Keywords:** Cyclophilin a, PPIase, CD147, cell proliferation, cell invasion, drug resistance, Inhibitor, cancer therapy

## Abstract

Cyclophilin A (CypA), a member of the immunophilin family, stands out as the most prevalent among the cyclophilins found in humans. Beyond serving as the intracellular receptor for the immunosuppressive drug cyclosporine A (CsA), CypA exerts critical functions within the cell via its *peptidyl-prolyl cis-trans isomerase* (*PPIase*) activity, which is crucial for processes, such as protein folding, trafficking, assembly, modulation of immune responses, and cell signaling. Increasing evidence indicates that CypA is up-regulated in a variety of human cancers and it may be a novel potential therapeutic target for cancer treatment. Therefore, gaining a thorough understanding of CypA’s contribution to cancer could yield fresh perspectives and inform the development of innovative therapeutic approaches. This review delves into the multifaceted roles of CypA in cancer biology and explores the therapeutic potential of targeting CypA.

## Introduction

Various types of cancer pose a global threat to human life, and their complexity represents a significant challenge for the development of new therapeutic strategies. Currently, a range of cancer therapies have been developed, including surgery, chemotherapy, radiation therapy immunotherapy, and targeted therapy.^1–4^ Among these, molecular targeted therapies are revolutionary therapeutics that interfere with specific molecules to inhibit cancer growth, progression, and metastasis. These therapies are useful either alone or in combination with standard chemotherapy agents. In fact, identifying an ideal target is essential for the successful development of molecular targeted therapies.^5,6^ Many studies have found several various biomarkers and signaling pathways that influence the malignant features of cancer cells, such as proliferation, anti-apoptosis, invasion, metastasis, angiogenesis, therapeutic resistance. Several molecular targeted therapies approved by the Food and Drug Administration (FDA) have demonstrated remarkable clinical success in treating various types of cancer, including breast, leukemia, colorectal, lung, and ovarian cancers.^7–14^ However, challenges like cancer metastasis, recurrence, heterogeneity, resistance to chemotherapy and radiation therapy can lead to treatment failure.^15^ Therefore, early detection and identification of novel therapeutic targets are crucial for the development of more effective treatments.

**Figure 1.**
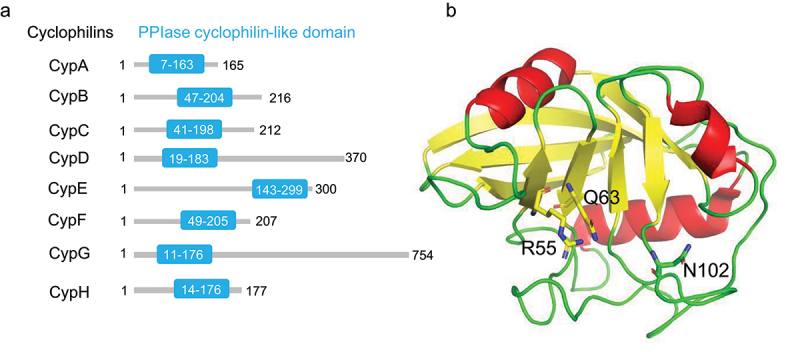
*PPIase* cyclophilin-type domain of human cyclophilins and 3D structure of CypA. (a) *PPIase* cyclophilin-type domain of cyclophilins. (b) The 3D structure of CyPA features with eight β-strands and two α-helices. The key catalytic residues, arginine 55 (R55), glutamine 63 (Q63) and asparagine 102 (N102), are labeled.

Cyclophilins are a conserved and ubiquitous family of proteins that are found in all living species, from *Escherichia coli* to *Homo sapiens*.^16^ Generally, these proteins possess *PPIases* activity that catalyzes the *trans* to *cis* isomerization of peptide bonds on proline residues.^17^ Indeed, owing to their enzymatic *PPIase* and/or binding activities, cyclophilins are involved in a wide variety of cellular processes, such as protein folding, trafficking, posttranslational modifications, protein assembly and cell signaling.^18^ To date, at least 18 known human cyclophilins have been identified, including Cyclophilin A (CypA), Cyclophilin B (CypB), Cyclophilin C (CypC), Cyclophilin D (CypD).^19^ All members of the cyclophilin family share a common domain known as cyclophilin-like domain (CLD), which is surrounded by domains unique to each protein. The CLD of different cyclophilin contains from 145 to 180 amino acid residues and has highly diversified sequences but displays a high level of structure conservation and retains a significant conservation of *PPIase* activity site (Table 1 and Figure 1a).^20,21^ CypA, the most abundant cyclophilin, was the first to be identified as a receptor for the immunosuppressive drug cyclosporin A (CsA) in cells, which binds the catalytic pocket of CypA with an affinity in the nanomolar range.^22^ Accumulating evidence shows that CypA not only participates in many biological processes, such as protein folding, immune regulation, cell growth, cholesterol metabolism, T-cell activation, cell signaling, but also involves the development of diseases such as inflammatory disease, viral infections and malignant tumors.^23–25^ Moreover, CypA is overexpressed in several types of cancer and promotes proliferation, anti-apoptosis, infiltration and metastasis, such as gastric cancer, breast cancer, colorectal cancer, liver cancer, lung cancer, melanoma. Consequently, CypA can be further investigated as a useful tool for early diagnosis, treatment and prevention of human cancers. This review mainly focuses on the critical role of CypA in cancer development and the therapeutic potential of targeted it in various cancer types.

**Table 1.** A list of the major forms of human cyclophilins.

Protein name	Gene name	Location in chromosome	Molecular mass (kDa)	Amino acids
Cyclophilin A (CypA)	PPIA	7p13	18	165
Cyclophilin B (CypB)	PPIB	15q21-q22	20	216
Cyclophilin C (Cyp C)	PPIC	5q23.2	33	212
Cyclophilin D (CypD)	PPID	4q31.3	40	370
Cyclophilin E (CypE)	PPIE	1p32	33	301
Cyclophilin F (CypF)	PPIF	10q22-q23	22	207
Cyclophilin G (CypG)	PPIG	2q31.1	88	754
Cyclophilin H (CypH)	PPIH	1p34.1	19	177

## Biological functions and structural characteristics of CypA

### CypA function

CypA, also known as peptidylprolyl isomerase A (PPIA), belongs to the cyclophilin family and possesses *PPIase* activity. It is primarily expressed in the cytosol. Increasing evidence showed that intracellular CypA can be secreted from various cell types, including vascular smooth muscle cells (VSMC), macrophages and endothelial cells (EC), in response to proinflammatory stimuli like hypoxia, infection or oxidative stress, in an autocrine or paracrine response.^40–42^ This secreted CypA facilitates various intercellular communication, intercellular responses and participates in numerous physiological or pathological processes. These include inflammation, cancer proliferation, metastasis, apoptosis, migration, matrix degradation, and generation of reactive oxygen species (ROS). In immune response, exogenous CypA has been shown to upregulate the expression of various cytokines and chemokines, including CXCL2, CXCL3, CXCL8, IL-1α and IL-1β.^43,44^) CypA can also interact with its cell surface receptor, CD147, to mediate chemotactic activities. For instance, CD147 facilitates the chemotactic effect of CypA on Jurkat T cells. CD147/CypA interaction may enable hepatoma cells to escape immune surveillance by T cells.^45^ Furthermore, the signaling axis formed by the Spike protein-CD147-CypA in SARS-CoV-2 is implicated in inducing a severe cytokine storm associated with COVID-19 cases. It is also suggested that CypA contributes to the cloaking of viral replication intermediates, representing an evasion strategy that obscures the detection of viral nucleic acids by the innate immune system.^46^ Additionally, CypA functioned as a critical positive regulator of RIG-I-mediated antiviral immune response. Deficiency of CypA impaired RIG-I-mediated type I IFN production. In inflammation, CypA can promote VSMC proliferation and migration, and activates matrix metalloproteinases (MMPs) like MMP-2 and MMP-9. It also induces EC apoptosis and the expression of adhesion molecules such as vascular cell adhesion molecule-1 (VCAM-1) and intercellular adhesion molecule-1 (ICAM-1). Notably, high CypA expression has been correlated with poor outcomes in patients with inflammatory diseases. In cells stimulated by extracellular CypA, pathways like extracellular signal-regulated kinase 1 and 2 (ERK1/2), nuclear factor kappa-B (NF-κB), protein kinase B (AKT), Jun N terminal kinase (JNK) and p38 mitogen-activated protein kinase (p38 MAPK) become activated.^47–50^ Recent studies highlight CypA’s overexpression in several cancer types, including gastric, breast, colorectal, liver, lung, and melanoma. This overexpression is associated with various pathological processes, such as tumor cell growth signaling, transcription factor regulation, apoptosis, metastasis, and drug resistance.^51–56^ CypA can alter the tumor microenvironment by fostering Th1 immune responses, modulating MMPs activity, and inducing proinflammatory cytokines like tumor necrosis factor alpha (TNFα) and interferon gamma (IFNγ). As a result, it might influence the early stages of tumor and metastasis development.^57^ Additionally, CypA plays a pivotal role in viral infections, either supporting or hindering viral replication in cells infected with various viruses like hepatitis C virus (HCV), severe acute respiratory syndrome-coronavirus-2 (SARS-CoV-2), human Immunodeficiency virus (HIV) or hepatitis B virus (HBV).^58,59^ Moreover, it has been suggested that CypA interacts with its cell membrane receptor CD147 and facilitates SARS-CoV-2 viral entry and replication.^24^ CypA also has roles in oxidative stress-mediated neurodegenerative diseases, rheumatoid arthritis (RA), aging, solidifying its position as a multifunctional protein involved in inflammatory disease and cancer progressiont (Table 2).^60–64^

**Table 2.** Major biological and pathological functions of CypA.

Functions	CypA targets	Biological/pathological effects	Reference
Protein folding	HIV-1 Gag Homo-oligomeric a7 neuronal nicotinic HCV NS5A	Promotion of both the formation and the infectivity of virions of HIV-1 Maturation of the homo-oligomeric CypA/NS5A complex promote HCV replication	26,27 28 29
Trafficking	CD147 Apoptosis-inducing factor (AIF) Asialoglycoprotein (ASGPR) Zinc-finger protein 1(Zpr1)	Transport to the plasma membrane Co-translocation to the neuronal nuclei to induce cell death after cerebral hypoxia-ischemia and ALS Distribution of ASGPR between the plasma membrane and the endosomal pool Promotion of nuclear export	30 31 32 33
T-cell activation	Interleukin-2 tryosine kinase (Itk)	Positive regulation of Th1 profile and inhibition of Th2 differentiation	34
Cell signaling	VCAM-1, E-selectin ERK1/2 JNK, NF-κB	Proliferation and migration of vascular smooth muscle cells Stimulation of VSMC growth Stimulation EC apoptosis and inflammation	35–37 36,38 39

### CypA Structure

From a structural perspective, CypA is encoded by the *peptidylprolyl isomerase A* (*PPIA*) gene located on chromosome 7 at position 7p13. This gene encodes a protein consisting of a single chain of 165 amino acids, with a molecular weight of approximately 18 kDa.^65^ To date, many structures of CypA, both in its free form and in complex with various interacting molecules (e.g., CsA, CsA analogues, substrate peptides, HIV-1 capsid protein), have been determined.^66,67^ It has been demonstrated that CypA has a cyclophilin-like domain typical of all the members of the cyclophilin family. Structurally, CypA has an eight-stranded antiparallel β-barrel structure, with two α-helix enclosing the barrel from either side.^68^ A hydrophobic core within the barrel, composed by residues such as Arginine 55 (R55), Phenylalanine 60 (F60), Glutamine 63 (Q63), and others, forms the active site of the *PPIase* enzyme. The detailed 3D structures of CypA active site have been elucidated (Figure 1b), identifying R55 and Lysine 82 (K82) as crucial for the catalytic activity of CypA-mediated cis/trans-isomerization.^69,70^ CsA can bind to the active site of CypA and interfering with *PPIase* activity of CypA. It has been demonstrated that the *PPIase* activity can catalyze isomerization of the peptide bond upstream of proline residues in proteins and it is required for protein folding, protein trafficking/molecular chaperoning, cell signaling and T cell activation.^67^ For instance, CypA can catalyze the prolyl cis-trans isomerization of proteins like interleukin-2 tyrosine kinase and cell signaling adaptor protein Crk, influencing their functions.^71–73^ In addition, the interaction between CypA and viral proteins plays an important role in the process of viral infection. It has been found that CypA facilitates influenza B viruses (IBV) replication by targeting IBV non-structural protein 1 (BNS1) and nucleoprotein (BNP).^74^ The HCV nonstructural protein 5A (NS5A) is an essential factor in the HCV life cycle which participates in both viral replication and assembly. CypA can directly interact with NS5A to promote the viral genome replication. The interaction between NS5A and CypA depends on both the proline residues in NS5A domain II and the *PPIase* active site of CypA.^75^ Additionally, mutations of amino acid residues in the active site of CypA abolish its ability to facilitate HCV replication. The H126Q CypA mutant, which diminishes CypA isomerase activity by more than 99% compared to wild-type CypA, fails to support HCV replication.^76–79^

### Membrane receptor for extracellular CypA

**Figure 2.**
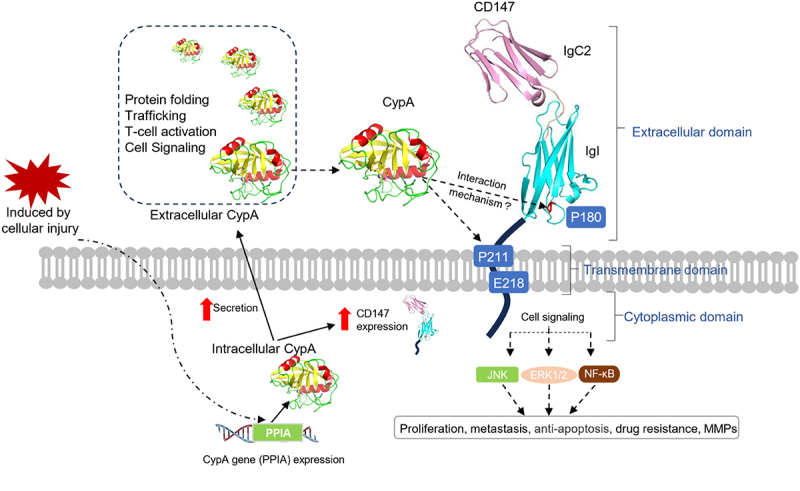
Structure, biological functions and interaction of CypA and CD147. Intracellular CypA is secreted to extracellular environments when induced by cellular injury, and it plays a vital role in protein folding, trafficking, T-cell activation and cell signaling. CD147, which serves as the membrane receptor of CypA, consists of two extracellular ig domains, IgC2 (colored in pink) and IgI (colored in cyan), a single transmembrane domain, and a short cytoplasmic domain. Extracellular CypA binds to the amino acid proline 180 (P180) of CD147 and induces signal transduction through subsequent interaction with proline 211 (P211). Major cancer-related signaling pathways regulated by CypA/CD147 are shown in the figure. Glutamic acid 208 (E208) of the CD147 transmembrane domain is also important for the signaling response.

Extracellular activities of CypA suggest existence of a receptor on target cells. Yurchenko et al. first identified CD147, also known as the extracellular matrix metalloproteinase inducer (EMMPRIN), as the membrane receptor for extracellular CypA. This molecule is released into the extracellular space by activated macrophages, smooth muscle cells and platelets, among others.^45,53,80^ Previous studies reported that CD147 is widely expressed in diverse cell types and participates in numerous physiological and pathological processes. These include cancer development, rheumatoid arthritis, plasmodium invasion and viral infections. CD147 may also act as a biomarker or therapeutic target for various human diseases, including cancer.^81,82^ It has been demonstrated that CD147 is highly expressed and may trigger the production or release of MMPs in surrounding mesenchymal and tumor cells, thus contributing to tumor invasion.^83^ Structurally, CD147 is a highly glycosylated transmembrane protein. It comprises a short cytoplasmic domain, a transmembrane domain, and an extracellular domain (IgI domain and IgC domain). These domains are essential for CypA-triggered signaling cascade that culminates in ERK1/2, p38 and NF-κB activation.^30,84,85^ Subsequent research has shown that CypA-induced chemotaxis and signaling pathways are achieved in two ways: one through its *PPIase* activity and the other through binding to CD147.^86^ Recent studies have indicated that both CypA and CD147 are upregulated in various types of cancers. The interaction between CypA and CD147 plays a crucial role in cancer development, affecting cell proliferation, metastasis, invasion, angiogenesis, anti-apoptosis and drug resistance. These processes can be blocked by either CypA inhibitors or CD147 antibodies, making the CypA/CD147 axis a potential target for the treatment of human cancers or inflammatory diseases.^87^ Therefore, understanding the molecular mechanism of CypA/CD147 interaction may have therapeutic value, especially in the treatment of cancer. However, the molecular mechanisms underlying the CypA/CD147 interaction remain poorly understood. Previous studies have shown that CypA binds to the amino acid Proline180 (P180) of CD147 and induces signal transduction through subsequent interaction with Proline 211 (P211). It has also been suggested that the Glutamic acid 218 (E218) is vital for signaling responses.^80^ However, Yand et al. found that the CypA/CD147 binding process might be regulated by Proline180-Glycine181 rather than P211 and that the amino acid Arginine 201(R201) is crucial for binding^30,88^ (Figure 2). Interestingly, Song et al. revealed that *PPIase* activity is not essential for CypA/CD147 interaction because CypA mutants lacking enzymatic activity but still binding to CD147 induced strong chemotaxis in the HL-60 cell line. The amino acids Histidine 70 (H70), Threonine 107 (T107) and Arginine 69 (R69) in CypA are key residues for interacting with CD147.^73^ However, until now, the complex structure of CypA/CD147 has not yet been resolved, and the mechanism of their interaction remains unclear.

### Roles of CypA in cancers

CypA is predominantly expressed in various human cells and was initially found to be up-regulated in hepatocellular carcinoma (HCC) in 1998.^89^ More recently, its overexpression has been reported in a wide range of cancers, including but not limited to breast cancer, ovarian cancer, gastric cancer, small cell lung cancer, non-small cell lung cancer (NSCLC), pancreatic cancer, colorectal cancer, cholangiocarcinoma, oral squamous cell carcinoma and melanoma.^53,90–98^ For instance, in lung cancer, Campa et al. reported that CypA levels were seven-fold higher than that in adjacent non-diseased lung tissues.^99^ Sustaining proliferative signaling, evading growth suppressors, resisting cell death, enabling replicative immortality, inducing angiogenesis, and activating invasion and metastasis are six biological capabilities of human cancer. It has been revealed that CypA participants in the whole cancer development process, including cell proliferation, invasion, metastasis, anti-apoptosis, and chemoresistance of multiple types of cancers, which suggested that a deeper understanding of CypA’s role in cancer biology could lead to new targeted therapies.^100^

### CypA promotes cell proliferation

CypA plays an important role in angiogenesis and endothelial cell proliferation. In small cell lung cancer, Yang et al. found that CypA is overexpressed. Exogenous CypA can promote H446 cell proliferation in a dose- and time-dependent manners which associated with its *PPIase* activity. Inhibition of CypA’s *PPIase* activity by CsA or the CypA-R55A mutated protein which lacks *PPIase* activity suppressed H446 cell proliferation. Researchers have investigated the signaling pathways involved in CypA-induced cell proliferation. It has been revealed that CypA activates ERK1/2 but not p38 and JNK signals in H446 cells.^90^ Additionally, in NSCLS, using quantitative reverse transcriptase polymerase chain reaction (qRT-PCR), it was found that an NSCLC cell line with characteristics of abnormal cells in the lung tissue, capable of invading and spreading to other parts of the body, had significantly higher levels of CypA expression compared to normal lung cell lines. CypA was shown to stimulate cell proliferation and increase cell invasion by regulating the activity of secreted MMP-9. When the expression of CypA is down-regulated by CypA-knockdown or RNA interference, cell proliferation, cell migration, invasion and MMP-9 secretion were significantly suppressed.^101^ In pancreatic cancer, CypA is also highly expressed and CypA interacts with the proline-containing peptide in the transmembrane domain of CD147, thereby stimulating human pancreatic cancer cell proliferation.^102^ This CypA-induced proliferation was substantially inhibited by an anti-CD147 antibody, suggesting mediation by CD147/CypA interaction. Further studies revealed that exogenous CypA stimulates the proliferation of pancreatic cancer cells by activating the ERK1/2 and p38 MAPK signaling pathways and by increasing the secretion of interleukin IL-5 and IL-17 in pancreatic cancer cells.^103^ In human cholangiocarcinoma tissues, overexpressed CypA enhances cholangiocarcinoma cell proliferation via direct interacting with CD147, along with activation of the ERK1/2 and p38 MAPK signaling pathways.^98^ In HCC, it has been found that CypA was higher expressed in HCC tissues than in adjacent tissues. CypA overexpression is associated with Tumor-Node-Metastasis (TNM) stage, which is a standardized system used in cancer staging to describe the extent of a cancer in the body. CypA also promotes the transition of the cell cycle from G1 to S phase in HCC cells.^104^ Taken together, inhibiting the expression of CypA can suppress cancer cell proliferation, invasion and metastasis, and CypA may act as a potential therapeutic target.

### CypA promotes metastasis

Metastasis is the primary cause of morbidity and mortality in cancer patients. Numerous studies have reported that CypA is associated with cancer metastasis and invasion across a variety of cancers. Local recurrence and distant metastasis frequently occur in patients with gastric cancer with high CypA expression.^105^ In pancreatic ductal adenocarcinoma (PDAC), CypA has been identified as a downstream gene of hypoxia-inducible factor (HIF)-1α. High expression of both genes is significantly associated with lymph node metastasis and advanced tumor stage.^91^ Guo et al. found a correlation between NSCLC cell migration and CypA levels in vitro, a finding that was consistent in mouse models. CypA knockdown resulted in the inhibition of NSCLC cell motility and invasion.^101^ Similarly, stable RNA interference of CypA in breast cancer and osteosarcoma cells led to reduced migratory capacity.^106^ Subsequent experiments revealed that CypA promotes NSCLC cell proliferation and metastasis through the activation of the p38 MAPK signaling pathway. In human endometrial cancer, Li et al. found that CypA knockdown by lentiviral shRNA (LV-shCypA) suppressed the migratory/invasive capacity of HEC-1-B cells, likely due to the down-regulation of focal adhesion signaling.^107^ Importantly, MMPs, key factors in the degradation of the extracellular matrix (ECM) in cancer, have been associated with cell invasion and migration. Increasing evidence suggests that MMPs are regulated by CypA in various cancer types. For example, CypA was found to enhance NSCLC cell invasion by regulating the activity of secreted MMP-9. Inhibition of CypA decreased MMP-9 activity.^53,101^ CypA silencing by RNA-interfering effectively suppressed the expression of MMP-2 and MMP-9, inhibiting metastasis and invasion in gastric cancer cells.^54,108^ Increased levels of MMP-2 and MMP-9 have also been observed in esophageal squamous cell carcinoma and are significantly correlated with the tumor differentiation and metastasis.^42^ In hepatocellular carcinoma, the level of CypA expression in two HCC-derived cell lines (MHCC97-L and MHCC97-H) was shown to correlate with their metastatic capability. Ectopic expression of CypA promoted cell adhesion, chemotaxis and lung metastasis *in vivo*, without affecting cell proliferation. Further studies revealed that CypA promotes HCC cell adhesion and metastasis through up-regulating MMP-3 and MMP-9.^54,104^ Thus, the current studies suggest that the up-regulation of CypA and MMPs is associated with various types of cancer, and CypA appears to play a vital role in cancer metastasis.

### CypA mediates anti-apoptosis

Apoptosis, the process of removing aged cells from the body, is an evolutionarily conserved and sequentially regulated. It plays an essential role in physiological and pathological conditions. Disruptions in this regulatory pathway can lead to a variety of diseases, like autoimmune diseases, neurodegenerative diseases and cancers.^109^ Anti-apoptosis is deemed crucial for tumor growth, and enhancing sensitivity to apoptosis induction is viewed as a promising strategy for cancer treatment.^110^ There is mounting evidence that CypA plays a vital role in tumorigenesis through its anti-apoptotic actions. Overexpression of CypA diminishes the responsiveness of apoptosis-related proteins to stressors such as hypoxia, cisplatin, and H_2_O_2._ This protective effect has been observed in various cancer cell lines, including DU145, HepG2 and HeLa cells, where it mitigates stress-induced apoptosis. Conversely, CypA knockdown using small interfering RNA exacerbates the effects of these stressors.^111^ Recent studies have discovered that CypA offers protective effects against hydrogen peroxide (H_2_O_2_)-induced oxidative injury and apoptosis in human lung carcinoma A549 cells. This protection is mediated through the activation of the phosphoinositide 3-kinase/protein kinase B/mammalian target of rapamycin (P13K/AKT/mTOR) signaling pathway.^112^ Inhibiting CypA activity induces cell death in both breast and gastric cancer cells.^113,114^ Similarly, CypA inhibition leads to significant apoptosis in endometrial carcinoma cells, accompanied by G1 arrest in cell cycle.^115^ In human gastric cancer cell lines AGS, silencing CypA triggers G2/M cell cycle arrest and apoptosis.^108^ Furthermore, CypA is significantly upregulated after radiation therapy in lung adenocarcinoma cells, and silencing of CypA greatly increases radiosensitivity, reinforcing cell apoptosis.^116^ These findings collectively suggest that CypA could be a pivotal target for modulating cancer cell apoptosis.

### CypA induces drug resistance

Drug resistance has been documented among many cancer therapeutics, including traditional chemotherapeutic agents, novel small-molecule inhibitors, and monoclonal antibodies. Although reports on CypA and drug resistance are still limited and preliminary, overexpression of CypA has been shown to induce resistance to hypoxia and chemotherapeutic agents such as cisplatin in cancer cells. Conversely, silencing CypA suppresses cancer cell viability, suggesting a potential role for CypA in drug resistance. Research has shown that liver cells stably expressing CypA (SK-Hep1-CypA) exhibit increased resistance to the anti-cancer drugs doxorubicin and vincristine. Moreover, accumulation of doxorubicin was found to be reduced in SK-Hep1-CypA cells. These findings imply that the elevated expression of CypA may be a factor in clinical resistance to chemotherapy in HCC.^117^ Additionally, upregulation of CypA was observed 12 h after high-dose treatments with cisplatin in HCC cells. When CypA was suppressed by CsA or sanglifehrin A (SFA), a synergistic effect with cisplatin was noted, enhancing cisplatin-induced apoptosis.^118^ Furthermore, CypA upregulation was noted in paclitaxel-resistant human endometrial cancer cells. Knocking down CypA reverses the paclitaxel resistance via suppression of MAPK kinase pathways.^115^ Peng et al. revealed that CypA is upregulated in chemoresistant colorectal cancer (CRC) samples. CypA was shown to reduce cellular reactive oxygen species levels and increase CRC cell survival when exposed to H_2_O_2_ and chemotherapeutics, mediated by a CypA-peroxiredoxin-2 (PRDX2) antioxidant mechanism. Targeting CypA with CsA displayed efficacy against chemoresistant CRC when paired with chemotherapeutics.^119^ Moreover, the overexpression of CypA led to the upregulation of drug metabolism and transport-related genes, such as IL-6, multidrug resistance-associated protein 2 (MRP2), multidrug resistance-associated protein 3 (MRP3), microsomal glutathione transferase 1(MGST1) and glutathione S-transferase zeta 1(GSTZ1). This upregulation contributed to drug resistance and diminished chemotherapy outcomes.^117^ The findings described suggest that CypA plays a role in chemoresistance and/or radioresistance, possibly by influencing the expression of drug resistance-related proteins, causing cell cycle arrest, and activating the MAPK signaling pathways, ultimately leading to unfavorable outcomes in cancer patients.

### CypA inhibitors and therapeutic targeting strategies

**Figure 3.**
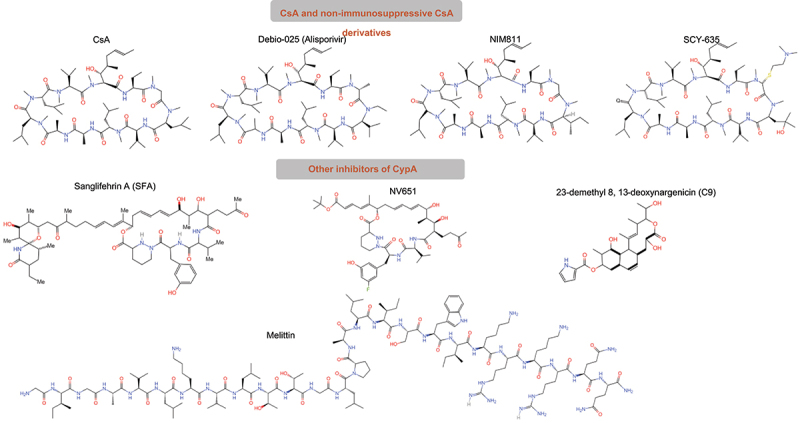
Chemical structure of compounds as CypA inhibitors. CsA and its derivatives, including alisporivir, NIM811, SCY-635 and CRV431 are on the top, C9, SFA, NV651 and Melittin are on the bottom.

Recently, targeting CypA for cancer therapeutics has recently begun to emerge because CypA overexpression is often found in various cancer types and it plays a role in malignant transformation. Agents that can potentially interfere with CypA or block the CypA/CD147 interaction may inhibit cancer development. Inhibitors of CypA and their potential targeting strategies are summarized in Table 3. Chemical structure of compounds of CypA inhibitors is shown in Figure 3.

**Table 3.** Major inhibitors of CypA and potential therapeutic strategies.

Inhibitors	Mechanism	Therapeutic Potential	Targeted Cancer	References
CsA	*PPIase* activity	Interference of CypA and CD147 binding, Induction of apoptosis	Breast cancer, Lung adenocarcinoma	113,120
Debio-025 (Alisporivir)	Crk signaling	Non-immunosuppressive analogue of CsA, Potent antitumor and antimetastatic activity, Enhancing of tumor immunogenicity and anti-PD-1 therapy	Breast cancer, Hepatitis C-hepatocellular carcinoma	121
Sanglifehrin A	*PPIase* activity	Binding to CypA with higher affinity than CsA	Glioblastoma multiforme	66,122
SCY-635, NIM811	NS5A	Non-immunosuppressive activity, Inhibition of hepatocarcinogenesis	Hepatitis C-hepatocellular carcinoma	22
NV651	*PPIase* activity	Non-immunosuppressive activity, inbibition of cell proliferation and tumor growth in vivo	Hepatocellular carcinoma	123
23-demethyl 8, 13-deoxynargenicin (C9)	MAPK signaling	Inhibition of proliferation, migration, invasion and angiogenesis	Gastric cancer	124
Melittin	MMP-9	Inhibition of metastasis	Breast cancer	125
RNA interference	*PPIase* activity	Inhibition of tumor growth, Enhancing of radiosensitivity	Lung adenocarcinoma	116
	NF-κB signaling	Inhibition of glioblastoma growth	Glioblastoma	126
Blocking interaction	CypA/CD147 interaction	Inhibition of tumor growth, metastasis, invasion	Hepatocellular carcinoma, breast cancer	100

### CsA

CsA, a well-studied CypA inhibitor that blocks *PPIase* activity, is an immunosuppressive, anti-inflammatory, antifungal, antitumor agent derived from the *fungus Tolypocladium inflatum*. As a lipophilic, cyclic undecapeptide, CsA has significantly improved the survival rates of patients and grafts post-solid-organ transplantation.^22,127,128^ Additionally, it is used to treat autoimmune diseases like psoriasis and rheumatoid arthritis.^129,130^ Growing evidence suggests CsA can inhibit tumor progression and viral infections. Notably, low micromolar concentrations of CsA have been shown to inhibit the replication of coronavirus, including SARS-CoV, MERS-CoV and SARS-CoV-2.^131^ Later, studies indicated that non-immunosuppressive CsA analogs also restrict coronavirus infections in cell culture, showing EC_50_ values compared to CsA. In the context of cancer, CsA not only can bind to CypA, inhibiting its *PPIase* activity but also disrupts CypA/CD147-related cell signaling and cellular functions by interrupting CypA/CD147 interaction. In breast cancer, CsA hampers cell proliferation by affecting glycolysis, particularly by downregulating the pyruvate kinase subtype M2 (PKM2).^113^ In lung adenocarcinoma, CsA is seen to suppress cancer cell growth by inducing apoptosis.^120^ Consistently, recent studies reveal CsA’s ability to boost docetaxel-induced apoptosis in human gastric carcinoma, suggesting its potential in cancer therapy.

### Non-immunosuppressive CsA derivatives

Due to the unwanted effects of CsA, including nephrotoxicity, its usage has been significantly limited. Agents that selectively inhibit CypA without causing side effects have been developed. Non-immunosuppressive CsA derivatives serve as one type of CypA inhibitors that lack immunosuppressive properties but maintains cyclophilin-binding activity. Examples include SCY-635, Debio-025 (Alisporivir), Debio-064, NIM811, NIM258 and CRV431.^87,132^ Among these, SCY-635, Debio-025, and NIM811 have been shown to alter certain replication steps of HCV and demonstrate clinical efficacy in treating HCV infection.^87,133–137^ CypA is a highly abundant protein and a host factor involved in HCV replication. It has been demonstrated that HCV NS5A protein which comprises three domains (named domain I, domain II and domain III) serves as a major viral ligand for CypA. Several studies have been demonstrated that CypA binds to proline-rich regions in domain II and III ofNS5A and the binding of CypA to NS5A is a prerequisite for HCV replication.^29,138^ CypA inhibitors block CypA/NS5A formation and destroy the CypA/NS5A complex.^79,139,140^ CsA is the first CypA inhibitor suppressing HCV replication. As CsA is a clinical immunosuppressant, it cannot be used as an anti-HCV agent.^141^ NIM258, a next-generation cyclophilin inhibitor synthesized through modification of CsA at P3-P4 positions, has also have shown excellent anti-HCV potency.^22,142^ Cyclophilin inhibitor SCY-635 can block HCV replication by disrupt CypA/NS5A interactions. Debio-064 is a structurally modified cyclosporine with approximately fivefold higher affinity for CypA than CsA. It can block arterivirus replication by interfering with viral RNA synthesis.^143^ To date, Debio-025, NIM-811 and SCY-635 have demonstrated safety and efficacy in patients with HCV in phase I and II studies.^135–137,144,145^ Non-immunosuppressive CsA derivatives have also been reported to inhibit cancer. Researchers have found that NIM811 and SCY-635 can inhibit hepatocarcinogenesis by disrupting CypA/NS5A interaction in HCV-induced HCC.^22^ Recent studies have shown that Debio-025 has antitumor and antimetastatic efficacy by suppressing the Crk signaling pathway in breast cancer. Furthermore, Debio-025, either alone or in combination with anti-PD-1 checkpoint inhibitors, significantly suppresses tumors in a triple-negative breast cancer model.^121^ CRV431, also named rencofilstat, is another nonimmune-suppressive cyclophilin inhibitor and is currently in Phase I clinical trials. It has been demonstrated that CRV431 not only inhibits HIV-1, HBV and HCV and liver damage including liver fibrosis and HCC, but also has the potential to treat liver fibrosis and cancer incidence.^146–150^

### Other inhibitors of CypA

SFA, a novel immunosuppressant isolated from *Streptomyces sp.*, strongly binds to CypA and inhibits its *PPIase* activity.^66^ When combined with cisplatin, SFA synergistically enhances cisplatin-induced apoptosis in C6 glioma cells.^122^ NV651, another new cyclophilin inhibitor based on the sanglifehrin scaffold but without immunosuppressant activity, shows potency in inhibiting cell proliferation in colorectal, liver and pancreatic cancer cell lines.^123^ NV651 has also been shown to decrease tumor growth in a xenograft mouse model.^151^ Subsequent studies have found that NV651 downregulates genes involved in cancer cell cycles and DNA damage repair pathways. Additionally, NV651, when combined with cisplatin，exerts a synergistic effect on cell viability and increases activation of apoptosis in HCC cells.^123^ 23-demethyl 8, 13-deoxynargenicin (C9), a novel nargenicin A1 analog, has been found to inhibit the growth of various cancer cell lines, including gastric, lung, liver, colon, brain, breast and cervical cancer.^108^ It also suppresses CypA expression and downregulates CD147-mediated MAPK signaling pathways, including JNK and ERK1/2, in human gastric adenocarcinoma cells.^108,124^ As a result, C9 suppressed the cell proliferation, migration, invasion and angiogenesis of gastric cancer cells. In lung cancer, C9 significantly hinders the proliferation and tumor sphere formation of NSCLC cancer stem cells (CSCs) and NSCLC-CSC-derived tumor growth in vivo by activating the intrinsic apoptotic pathway. Notably, C9 suppressed the expression of major CSC markers, such as CD44, integrin α6, through the dual downregulation of CypA/CD147 and EGFR in NSCLC CSCs.^152^ In addition, it has been found that C9 decreased in vitro AGS gastric cancer cell-induced angiogenesis of human umbilical vein endothelial cells (HUVECs) by blocking hypoxia-inducible factor-1α (HIF-1α) and VEGF expression in AGS cells.^108^ Small interfering RNA-based CypA knockdown and blocking interaction of CypA/CD147 could also be an effective therapeutic approach against cancer, such as human glioblastomas and lung adenocarcinoma cells.^108,116,126,153^ Melittin, a polypeptide containing 26 amino acid residues, has been reported to induce cell apoptosis in various of cancer, such as lung cancer, HCC, breast cancer, and prostate cancer.^154–157^ Previous studies have indicated that melittin inhibits CypA in macrophage cells and downregulates the invasion level of breast cancer MCF-7 cells in a dose-dependent manner by downregulating CD147 and MMP-9 through inhibition of CypA expression.^125^ Therefore, CypA represents a potential anticancer target, and agents that target CypA or the CypA/CD147 interaction can suppress cell proliferation, metastasis, and drug resistance of cancer.

## Conclusion and perspectives

Despite remarkable advances in cancer diagnostic and diverse new treatment strategies have been developed, the survival rate of patients with advanced or metastatic cancer remains low due to treatment resistance and recurrence. Consequently, cancer remains a significant threat to human health. The urgency to identify new drug targets and devise innovative therapeutic strategies for cancer treatment cannot be overstated. Based on the current knowledge reviewed above, CypA is a multifunctional molecule that acts as a key mediator in numerous physiological and pathological conditions. Over the past few years, there has been mounting evidence pointing toward CypA’s involvement in an array disease, encompassing viral infection, inflammatory diseases (such as rheumatoid arthritis, periodontitis), cardiovascular disease, oxidative stress-mediated neurodegenerative diseases, immunodeficiency disease, sepsis, aging, and notably, cancer. This review highlights the critical roles of CypA in cancer cell proliferation, invasion/metastasis, anti-apoptosis, and drug resistance while also spotlighting the therapeutic potential of targeting CypA signaling and the promise of CypA as a drug target. Elevated CypA expression is clearly contributory to pathological conditions and cancer development, with CypA knockdown often resulting in reduced tumor growth or invasion/metastasis. Notably, high CypA expression correlates with poor cancer patient outcomes. This suggests that CypA has significant roles in various cancers, positioning CypA as a potential biomarker for diagnostics, treatment, and prognostics across different cancer types.

It is worth noting that while many studies have reported on the involvement of CypA in cancer progression, the pathogenic roles of CypA and its molecular mechanisms in most cancers remain largely uncharted and merit further clarification. Additionally, the role of CypA in cancer likely involves a multifaceted interplay of proteins and pathways, such as the interaction with its membrane receptor, CD147. membrane receptor for CypA, CD147. The CypA/CD147 interaction, associated with cancer progression, is still entirely understood and warrants additional research. From a clinical standpoint, multi-drug resistance (MDR) is a common challenge in treating numerous malignant tumors, making CypA a potential focal point for clinical interventions. Compounds that either selectively inhibit CypA or block its binding to its receptor could be an avenue for cancer treatment exploration. Hitherto, while various inhibitors have been extensively researched, none have been deemed ideal for clinical use, including CsA. Therefore, there is a pressing need for the development of inhibitors, particularly those that selectively target CypA without eliciting immunosuppressive effects. The long-term usage of CsA in organ transplant recipients has been linked to several side effects, with nephrotoxicity being a significant concern. This hampers the broader application of the drug in treating inflammation and other immune-related disorders. Although non-immunosuppressive derivatives of CsA show potential for treating infections like HIV-1 and HCV, questions surrounding their nephrotoxicity and their role in cancer treatment still remain. In summary, the existing research robustly indicates CypA’s potential as a predictive biomarker for cancer diagnosis and as a therapeutic target for drug development. Utilization of a clinically safe CypA inhibitor or that block binding of CypA to its receptor can be considered as an alternative therapeutic approach for future management of cancer, but it is still a long way to go.

## Author‘s contributions

Shujuan Jin, Mengjiao Zhang and Xiaoting Qiao conceived the article and performed the literature reviews. Shujuan Jin wrote the main manuscript text and prepared the manuscript. Shujuan Jin, Mengjiao Zhang and Xiaoting Qiao critically revised and improved the manuscript. All authors read and approved the final manuscript.
